# Probing Non-Equilibrium Pair-Breaking and Quasiparticle Dynamics in Nb Superconducting Resonators Under Magnetic Fields

**DOI:** 10.3390/ma18030569

**Published:** 2025-01-27

**Authors:** Joong-Mok Park, Zhi Xiang Chong, Richard H. J. Kim, Samuel Haeuser, Randy Chan, Akshay A. Murthy, Cameron J. Kopas, Jayss Marshall, Daniel Setiawan, Ella Lachman, Joshua Y. Mutus, Kameshwar Yadavalli, Anna Grassellino, Alex Romanenko, Jigang Wang

**Affiliations:** 1 Ames National Laboratory, U.S. Department of Energy, Ames, IA 50011, USA; joongmok@iastate.edu (J.-M.P.); rkim@iastate.edu (R.H.J.K.); 2 Department of Physics and Astronomy, Iowa State University, Ames, IA 50011, USA; ianchong@iastate.edu (Z.X.C.); shaeuser@iastate.edu (S.H.); rkchan@iastate.edu (R.C.); 3 Fermi National Accelerator Laboratory, Batavia, IL 60510, USA; amurthy@fnal.gov (A.A.M.); annag@fnal.gov (A.G.); aroman@fnal.gov (A.R.); 4 Rigetti Computing, Berkeley, CA 94710, USA; ckopas@rigetti.com (C.J.K.); jayss@rigetti.com (J.M.); danielosetiawan@gmail.com (D.S.); elachman@rigetti.com (E.L.); jmutus@rigetti.com (J.Y.M.); kyadavalli@rigetti.com (K.Y.)

**Keywords:** ultrafast pump–probe, superconductivity, superconducting quantum computer, non-equilibrium quasiparticles, superconducting radio frequency (SRF)

## Abstract

We conducted a comprehensive study of the non-equilibrium dynamics of Cooper pair breaking, quasiparticle (QP) generation, and relaxation in niobium (Nb) cut from superconducting radio-frequency (SRF) cavities, as well as various Nb resonator films from transmon qubits. Using ultrafast pump–probe spectroscopy, we were able to isolate the superconducting coherence and pair-breaking responses. Our results reveal both similarities and notable differences in the temperature- and magnetic-field-dependent dynamics of the SRF cavity and thin-film resonator samples. Moreover, femtosecond-resolved QP generation and relaxation under an applied magnetic field reveals a clear correlation between non-equilibrium QPs and the quality factor of resonators fabricated by using different deposition methods, such as DC sputtering and high-power impulse magnetron sputtering. These findings highlight the pivotal influence of fabrication techniques on the coherence and performance of Nb-based quantum devices, which are vital for applications in superconducting qubits and high-energy superconducting radio-frequency applications.

## 1. Introduction

Most modern quantum computers are built on superconducting (SC) transmon qubits incorporating Josephson junctions (JJs) made of aluminum oxide between two aluminum (Al) electrodes [1,2,3]. Niobium (Nb) is widely used for RF resonators due to its high critical temperature and well-established lithographic patterning, which enables the control, readout, and coupling of multiple qubits in superconducting quantum circuits [4,5,6,7,8,9]. To achieve scalable multi-qubit systems, qubit states require long relaxation times ($T_{1}$) and dephasing times ($T_{2}$), typically greater than 100 μs [10,11,12]. Significant efforts have been made to study pair-breaking and loss mechanisms to extend $T_{1}$ and $T_{2}$ [1,13,14]. Microwave dielectric losses at metal–dielectric interfaces, such as at Nb–substrate boundaries, have been one of the limiting factors for qubit lifetimes. Intrinsic losses in bulk materials should theoretically permit lifetimes exceeding 1 ms. Despite being a type II superconductor with a high critical temperature $T_{c}$ = 9.3 K, Nb used in microwave circuits for transmon qubits has been identified as a potential source of decoherence. Therefore, understanding the decoherence and loss mechanisms in Nb superconducting states is crucial for improving qubit performance. Shorter-than-expected $T_{1}$ and $T_{2}$ times are often attributed to two-level system (TLS) losses below 1 K and non-equilibrium quasiparticle (QP) generation above 1 K, stemming from factors such as nonuniform surface morphology, defects, native Nb oxide, and ionizing radiation [15,16,17,18].

**Figure 1:**
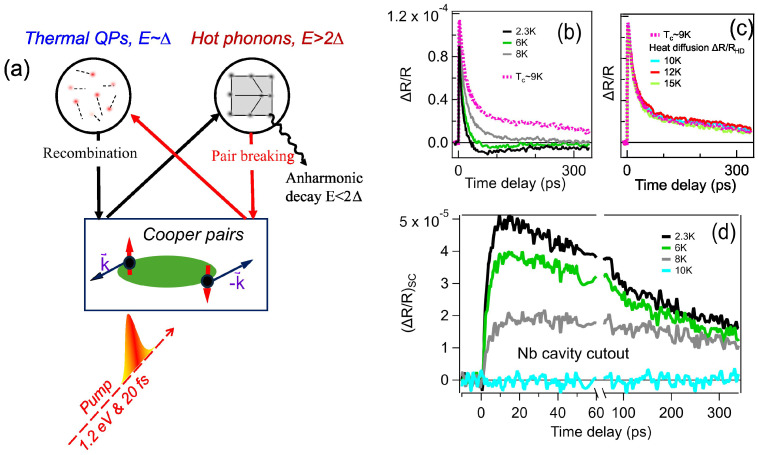
Temperature-dependent photoinduced reflectivity change ΔR/R of Nb SRF cavity cutout sample. (**a**) Schematic diagram of pair-breaking mechanism in superconducting Nb with ultrafast optical pump having photon energy ℏω≫2Δ. Thermal QPs are generated by high-frequency phonon via pair breaking. (**b**,**c**) Measured pump–probe ΔR/R dynamics for 2 mm thick Nb cavity cutout at 2.3 K, 6 K, and 8 K SC states and at 10 K, 12 K, and 15 K normal states above Tc. (**d**) Superconducting $\Delta R/R_{SC}$ signals are obtained with subtraction from average normal-state data.

The ultrafast pump–probe technique using femtosecond (fs) laser pulses is a powerful and versatile method for investigating non-equilibrium Cooper pair breaking and quasiparticle dynamics in superconductors. Transient signals measured after ultrafast excitation provide exclusive insight into the out-of-equilibrium processes arising from the conversion between the superconducting condensate and quasiparticles (QPs). In our fs-resolved transient reflectivity scheme, weak 1.2 eV laser pulses break a small fraction of Cooper pairs in the Nb samples. This non-thermal depletion of the superconducting condensate induces a noticeable change in reflectivity at the probe frequency, as illustrated schematically in Figure 1a. This scheme allows us to measure the transient changes in reflectivity as a function of the time delay, temperature, and magnetic field in Nb superconducting states. According to the well-established Rothwarf–Taylor (R-T) model [19], $\Delta R_{T\to 0}/\Delta R\left(T\right)-1$ directly corresponds to the thermal quasiparticle density $n_{T}$ in the weak perturbation regime, where the depletion of superfluid density $\Delta n/n_{s}\ll 1$ [20].

To differentiate the quasiparticle (QP) recombination process from electron and phonon temperature changes driven by laser heating, experiments are conducted at two temperature ranges: above and below Tc. The superconducting response is obtained by isolating the low-temperature signal from the high-temperature one [21]. For instance, optical pump–probe experiments on Pb [22] show that femtosecond laser pulses rapidly induce electronic transition, followed by a fast decay (∼1 ps) as energy transfers to QPs and high-frequency phonons (HFPs). HFPs, which have energies exceeding the superconducting gap, break Cooper pairs until majority of energy dissipates through electron–phonon interactions or thermal diffusion. In this weak perturbation regime, the photoinduced QP density is much smaller than the thermal-equilibrium QP density. To maintain a weak perturbation, the laser fluence should be kept low (<10 μJ/cm^2^) [21,22,23,24] to avoid breaking a large number of Cooper pairs. Initially, photo-excited hot electrons equilibrate rapidly, followed by a slower decay of HFPs. Once the photoinduced QPs reach a quasi-equilibrium state with HFPs within a few picoseconds, a slower decay process ensues, driven by HFP anharmonic decay and bi-molecular QP recombination within the phonon bottleneck regime. The dynamic pair-breaking and recombination process induced by the ultrafast laser must be modeled as a non-equilibrium state, in which pair recombination is coupled with high-frequency phonons and Cooper pairs [19,20,25,26].

Moreover, a magnetic field is anticipated to significantly influence the non-equilibrium QP generation and decay processes through magnetic vortex formation and field penetration, offering a valuable approach to assessing the robustness of superconducting coherence. However, ultrafast pump–probe experiments exploring the magnetic field dependence of non-equilibrium superconducting dynamics have not yet been conducted on Nb superconductors. Despite several pump–probe studies on QP dynamics in unconventional superconductors such as cuprates [20,27,28] and pnictides [29,30,31], fs studies of equilibrium QP dynamics on conventional BCS-type SCs are relatively rare.

In this article, we conducted an in-depth investigation into the non-equilibrium QP generation and relaxation dynamics in superconducting Nb sourced from radio-frequency cavities, as well as various Nb resonator films fabricated using deposition techniques such as DC sputtering and high-power impulse magnetron sputtering (HiPIMS). We obtained QP densities from pump–probe signals under different temperature and magnetic field conditions. Our results reveal both shared characteristics and distinct differences in the temperature- and magnetic-field-dependent behavior of Nb samples designed for SRF and qubit applications. Significantly, fs-resolved QP relaxation under an applied magnetic field demonstrated a clear correlation between the QP density and the quality factor of Nb thin-film resonators. These findings provide valuable insights into non-equilibrium QP dynamics in Nb materials and lay the groundwork for systematic measurements that could guide strategies to develop highly coherent quantum devices.

## 2. Materials and Methods

Nb thin films fabricated with different growth methods and bulk Nb from a superconducting radio-frequency (SRF) cavity used in the experiments are summarized in Table 1. Sputter-coated Nb thin-film samples of thickness t = 175 nm were grown by HiPIMS, DC high-power sputtering, and DC low-to-high (DC LH)-power sputtering on intrinsic Si (001) wafers at Rigetti Computing. The deposition rate for HiPIMS was ∼5.1 nm/min, and the DC high-power deposition rate was ∼25 nm/min. For the DC LH sample, the low-power deposition rate was 5.3 nm/min to 30 nm thickness, followed by 145 nm deposition with high power. The average grain sizes of sputtered samples were 44 nm for HiPIMS, 69 nm for DC high, and 65 nm for DC LH. A thick t = 2 mm SRF sample was obtained as an SRF cavity cutout from Fermi National Accelerator Laboratory. The polycrystalline SRF sample has a large average grain size of about 50 μm, compared to 44–69 nm for sputter-coated samples. Detailed sample information and growth methods are described elsewhere [32,33]. The SRF cavity cutout sample was mechanically polished for optical measurement. To avoid oxidation, the sample was polished with oil-based diamond powders, rinsed with isopropyl alcohol, and then dried with nitrogen gas. Sputter-coated thin-film samples were cut to 10 × 10 mm^2^ with a diamond pen from a 3-inch wafer. All samples were kept in a dry box before mounting in a vacuum cryostat to avoid contamination and moisture absorption. The thermal conductivity of Nb itself is ∼100 kW/m·K, much higher than Si ∼ 80 W/m·K near Tc.

The quasiparticle density and decay dynamics induced by ultrafast excitation are influenced by intrinsic processes, such as hot phonons and the photon bottleneck effect, as well as extrinsic processes, including pinning and pair breaking caused by defects and grain boundaries. In the studied multi-crystallized samples, extrinsic processes dominate. As shown in Table 1, grain size, defective boundaries, and other impurities are strongly correlated with the growth methods, significantly impacting the superconducting and transport properties of the samples, such as T*_c_*, RRR values, and grain topology. Consequently, the thermalized QP density and decay dynamics are closely linked to the growth methods, which produce distinctly different grain boundaries and pinning centers. Additionally, in the mixed state of type II superconductors, vortex cores formed under a magnetic field can trap QPs and influence their relaxation dynamics. The formation and pinning of these vortices are strongly influenced by grain boundaries and impurities, underscoring the pivotal role of growth methods in shaping QP density and dynamics. These effects will be investigated in detail below using ultrafast spectroscopy.

Our ultrafast pump–probe measurements were performed using a femtosecond pulsed laser with a 7 Tesla dry cryostat. The experimental setup employed a normal reflection geometry for pump–probe spectroscopy [34]. A 1250 nm laser output was split into two amplified arms via optical fibers, generating 20 fs light pulses at 1500 nm. One arm served as the pump beam, while the other functioned as the probe beam at 750 nm, generated through second-harmonic generation. The pump and probe beams were overlapped using a dichroic beam combiner and focused with a 100 mm focal length lens in a collinear geometry. Samples were mounted facing the top window in the cryostat, with the magnetic field applied perpendicular to the sample surface.

**Figure A1:**
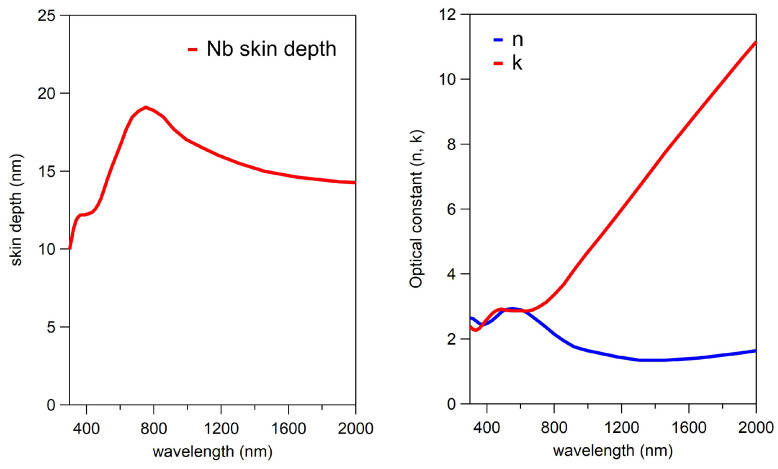
(**left**) Optical skin depth of Nb with wavelength. (**right**) Real and imaginary parts of optical constant n+ik of Nb.

A silicon balanced detector paired with a lock-in amplifier was used to detect reflectivity changes, with a 40 kHz mechanical chopper placed in the pump beam path. The temporal overlap of the pump and probe beams was controlled via a motorized linear delay stage in the pump path. The reflected probe signal was directed to the balanced detector, with a reference beam of equal optical path length focused onto the detector’s reference channel to cancel out noise. A 900 nm short-pass filter was used to block the 1500 nm pump beam entirely. The laser’s focal point diameter was approximately 100 μm, with pump fluences ranging from 1 to 7 μJ/cm^2^ for fluence dependence and the probe fluence kept at around ∼0.3 μJ/cm^2^ to ensure minimal disturbance. A common pump fluence of 3 μJ/cm^2^ was used for most measurements to achieve both a high signal-to-noise ratio and operation within the weak perturbation regime. The optical skin depth of Nb, calculated as δ=λ/4πk, was found to be 19 nm at 750 nm and 15 nm at 1.5 μm based on optical constants [35], as shown in Appendix A Figure A1. The analysis of QP dynamics under varying magnetic fields, along with a comprehensive comparison of the ultrafast superconducting behavior across different Nb samples, will be discussed in detail in the following sections. Additionally, the thin-film sample’s residual resistivity ratio (RRR = R(290 K)/R(10 K)) and the power-dependent quality factor $Q_{i}$ were measured using fabricated Nb resonators at the qubit operating frequency of 5 GHz.

## 3. Results and Discussion

Figure 1b,c present the representative pump–probe responses of the photoinduced reflectivity change, ΔR/R, at 2.3 K, 6 K, and 8 K in the superconducting (SC) state, as well as in the normal state above the $T_{c}$ of the Nb cavity cutout sample. Both ΔR/R signals exhibit a sharp rise time of approximately 3 ps, followed by a slower decay lasting over 300 ps. The rapid increase is attributed to QP generation from initial pair breaking by the laser pulse, followed by electron–phonon scattering, while the slower decay is mainly due to high-frequency phonon relaxation and thermal diffusion. Thermal diffusion primarily occurs vertically rather than laterally, as the laser focal spot, measuring tens of micrometers, is significantly larger than the optical skin depth (∼15 nm). In Figure 1b, the ΔR/R signal includes both the photoinduced QP and HFP generation and the thermalization process, as well as the vertical thermal diffusion. The latter components of ΔR/R shown in Figure 1c exhibit minimal temperature dependence for small temperature changes (≤10 K). Subtracting ΔR/R from the average normal-state ΔR/R isolates the photoinduced QP dynamics in the SC state $\Delta R/R_{SC}$, as shown in Figure 1d. This $\Delta R/R_{SC}$ component, absent above the critical temperature, shows both a slower rise time of ∼5 ps and a slower decay compared to the dynamics observed prior to subtraction. Our data clearly demonstrate the capability to independently measure the dynamics of quasiparticle generation/relaxation from the SC condensate and hot-phonon thermal diffusion processes. This methodology was subsequently extended to investigate additional Nb samples fabricated through various techniques.

A theoretical QP dynamic model was developed for the non-equilibrium states measured in Figure 1d after laser excitation [19,20,25,26]. Rothwarf and Taylor (R-T) proposed two coupled equations to relate the QP recombination and non-negligible high-frequency phonons created by the initial energetic QP during laser excitation. The coupled photoinduced HFP pair breaking and bi-molecular QP recombination process governs the long QP decay dynamics in the phonon bottleneck regime [19], as shown in Figure 1d. In the R-T model, phonons generated from quasiparticle recombination have a high likelihood of being absorbed in a subsequent pair-breaking process. This high-frequency phonon can then break a Cooper pair, creating two additional QPs, as illustrated in Figure 1a. The recovery of superconductivity results from the decay of photoinduced non-equilibrium QPs. This hot-phonon-mediated pair-breaking and recombination process has a longer lifetime than the intrinsic hot-electron decay. QP dynamics also require a detailed balance between phonon generation, decay, and the phonon-driven pair-breaking process [25]. Since the laser is focused on a small area of the sample, hot-phonon diffusion should also be considered to analyze the $\Delta R/R_{SC}$ component for non-equilibrium QP dynamics in SC states.

**Figure 2:**
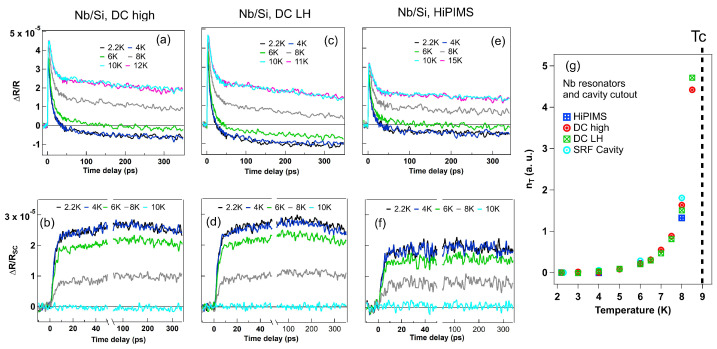
Temperature-dependent photoinduced reflectivity change ΔR/R of Nb thin films and QP density of Nb samples. (**a**,**c**,**e**) Temperature-dependent ΔR/R of Nb thin-film samples from T = 2.2 K to T = 15 K for top figures. (**b**,**d**,**f**) Superconducting state contributions in $\Delta R/R_{SC}$ components subtracted from average normal stage values for bottom figures. (**g**) Temperature-dependent equilibrium QP densities of HiPIMS, DC high power, DC LH power, SRF cavity samples. Pump fluence is set to be 3.0 μ J/cm^2^.

Next, we present the ultrafast dynamics of quasiparticles and phonons in thin-film Nb resonators. Figure 2a,c,e show temperature-dependent ΔR/R, and Figure 2b,d,f show subtracted superconducting components $\Delta R/R_{SC}$ from normal-state average traces of DC high-power, DC LH-power, and HiPIMS Nb samples. We emphasize two key points. First, the SRF cavity sample exhibits significantly better thermal diffusion compared to the thin-film samples, which accounts for the faster QP decay times (Figure 1d) observed in the Nb cavity cutout sample compared to Nb thin films. The SRF cavity sample is polycrystalline with an average grain size of 50 μm, whereas the HiPIMS Nb thin film has a grain size of ∼10 s of nm. Smaller grains lead to increased grain boundaries and defects, which act as QP pinning centers that slow recombination. Moreover, HFP diffusion within the optical depth is significantly more efficient in the SRF cavity sample due to its larger grain size. Second, the photoinduced superconducting $\Delta R/R_{SC}$ signals in Figure 2b,d,f approach zero as the temperature nears $T_{C}$. The signal amplitude directly correlates with QP generation, while the time dependence reflects the QP relaxation dynamics. The temperature-dependent thermal-equilibrium QP density $n_{T}$ is estimated from $n_{T}\left(T\right)\propto Q\left(T\to 0\;K\right)/Q\left(T\right)-1$, where Q(T) is the peak intensity in $\Delta R/R_{SC}$ in Figure 2b,d,f. Q(T→0K) is obtained from the lowest temperature value at 2.2 K. The thermal-equilibrium QP density $n_{T}$ measured in Figure 2g agrees well with Equation (1) [20],

(1) $$n_{T}\left(T\right)=n\left(0\right)\sqrt{2n\Delta_{SC}k_{B}T}\;e^{-\Delta_{SC}/k_{B}T},$$

where n(0) is the electronic density of states in the unit cell and $2\Delta_{SC}$ is the superconducting energy gap. And, the phonon density $N_{T}$ at thermal equilibrium is shown in Equation (2) [20].

(2) $$N_{T}\left(T\right)=\left(36\nu \Delta_{SC}^{2}T\right)/\omega_{D}^{3}\;e^{-2\Delta_{SC}/k_{B}T},$$

where $\omega_{D}$ is the Debye energy, ν is the number of atoms in the unit cell. The initial photoinduced QP density Δn and excited phonon density ΔN are small compared to $n_{T}$ and $N_{T}$ in the weak perturbation limit, i.e., $\Delta n/n_{s}\ll 1$.

**Figure A2:**
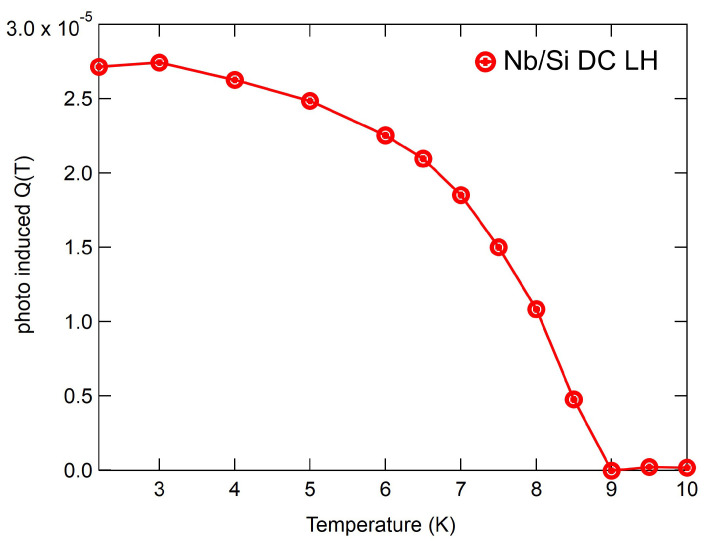
Peak value of photoinduced QP density Q(T) of Nb DC low–high sample.

Because the incident photon energy ∼ 1 eV is much larger than the SC gap 2Δ∼3 meV, the initial energetic QP after laser excitation on the sample generates an HFP. After energetic QP dissipation in a few ps, QP generation and recombination are driven by balanced states between HFP pair breaking and QP recombination, as shown in Figure 1a’s schematic, i.e., the strong phonon bottleneck region. Thermal-equilibrium QP densities, $n_{T}$, derived from measurements, exhibit an exponential increase with temperature, as shown in Figure 2g. Additionally, in the SC state, the high-frequency phonon density dissipates heat through electron–phonon interactions and diffusion. The pump–probe signal, ΔR/R, in SC states includes temperature-independent contributions below T*c*, such as rapid thermal and carrier diffusion [30]. The SC contribution, $\Delta R/R_{SC}$, is again isolated by subtracting the normal-state signal measured above Tc, which is proportional to Q(T). As shown in Appendix A Figure A2, this quantity diminishes as the temperature approaches Tc.

Pump–probe experiments reveal that thin-film samples with smaller grain sizes exhibit higher quasiparticle densities, longer QP relaxation times back to the condensate, and lower Q factors. These effects can be attributed to quasiparticles becoming trapped in grain boundaries and defect centers, which inhibit the reformation of the condensate state. Samples with smaller grains inherently have larger grain boundary areas and potentially a higher density of defects. These trapped quasiparticles undergo a delayed recombination process back into the condensate, thereby extending the relaxation time.

**Figure 3:**
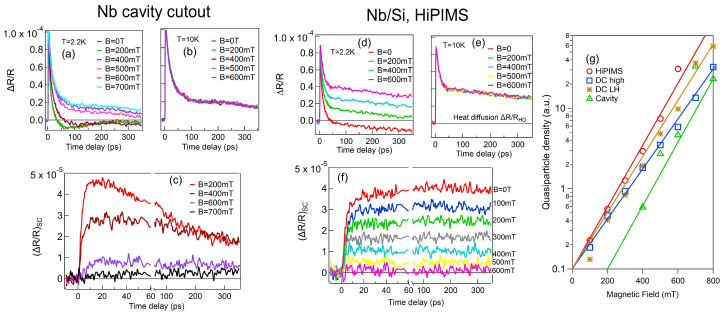
Magnetic-field-dependent ΔR/R of Nb samples. (**left**) Nb SRF cavity cutout ΔR/R: (**a**) low temperature at T = 2.2 K, (**b**) normal state at T = 10 K, (**c**) SC component $\Delta R/R_{SC}$ subtracted from average 10 K value. (**middle**) Thin-film Nb HiPIMS ΔR/R: (**d**) low temperature at T = 2.2 K, (**e**) normal state at T = 10 K, (**f**) $\Delta R/R_{SC}$ subtraction from average 10 K value. (**g**) Magnetic-field-dependent thermal-equilibrium QP densities $n_{T}\left(B\right)$ of HiPIMS, DC high, DC LH, and SRF cavity samples. Pump fluence is set to be 3.0 μJ/cm^2^.

Figure 3a,b show the magnetic field dependence of ΔR/R on the Nb SRF cavity at 2.2 K and 10 K. The results demonstrate a strong magnetic field dependence of ΔR/R at 2.2 K, while no clear field dependence is observed at 10 K. The extracted SC component $\Delta R/R_{SC}$ of the SRF cavity reveals a distinct magnetic field dependence in QP generation, with the majority of Cooper pairs breaking at B > 700 mT, as shown in Figure 3c. The ΔR/R behavior of the HiPIMS sample, as shown in Figure 3d,e, resembles that of the SRF cavity samples. The subtracted superconducting component, $\Delta R/R_{SC}$, of the HiPIMS sample also displays magnetic-field-dependent QP dynamics, though a majority of Cooper pairs break at a lower magnetic field of B∼600 mT, as shown in Figure 3f. Intriguingly, there is a clear difference in the $\Delta R/R_{SC}$ components under the external magnetic field between the SRF cavity (Figure 3e) and Fe resonator (Figure 3f) samples. First, HiPIMS has a much longer relaxation time and also requires a lower magnetic field to break the majority of Cooper pairs, as shown in Figure 3f, compared to the cavity cutout sample in Figure 3c. Other DC sputter samples show similar trends to HiPIMS. Second, the thermalized QP densities $n_{T}\left(B\right)$s extracted from photoinduced $\Delta R/R_{SC}$ signals are plotted together for HIPIMS, DC high, DC LH, and the SRF cavity, as shown in Figure 3g with guided lines. The SRF cavity sample exhibits the lowest quasiparticle population and the most robust superconducting properties, showing strong resistance to external magnetic fields. Notably, a distinct threshold magnetic field is required to induce a significant quasiparticle population. The quasiparticle density generation behavior in the SRF cavity sample is succeeded in resilience by the DC high, DC LH, and HiPIMS samples, exhibiting progressively higher densities in that order. Moreover, the QP signals of these thin-film samples decrease steadily from 0 to 400 mT without a threshold, whereas the SRF cavity sample shows an uneven reduction in QP signals, generating QPs only at sufficiently high fields. Among the thin-film samples, the magnetic-field-induced quasiparticle density exhibits an increasingly steep slope in the order of DC high, DC LH, and HiPIMS. These findings underscore the SRF cavity sample’s superior superconducting properties and high tolerance to pair-breaking perturbations, followed by the DC high, DC LH, and HiPIMS samples, making them progressively suitable for applications requiring highly coherent quantum devices.

We can attribute these differences to the grain heterogeneity and magnetic field penetration into the sample. The SRF cavity requires a stronger magnetic field to break SC states due to the large grains that are more robust in external magnetic field penetration. The low QP density in $n_{T}\left(B\right)$ indicates that a strong magnetic field is needed to penetrate the sample. Additionally, DC samples tend to produce films with larger grain sizes compared to HiPIMS ones. This difference in our observations arises due to the distinct deposition processes and energy distributions in the two techniques [32]. In general, large-grained samples inherently have fewer grain boundaries as flux pinning centers. Thus, it is expected that a magnetic field can easily penetrate the HiPIMS sample. Therefore, among sputter-coated samples, the DC high sample is robust in magnetic field penetration. Assuming that thermal diffusion in sputter samples is not much different due to the similar overall thickness of the Nb and interface layers, one expects that the magnetic field can penetrate more easily and break more QPs in small-grain thin films.

**Figure 4:**
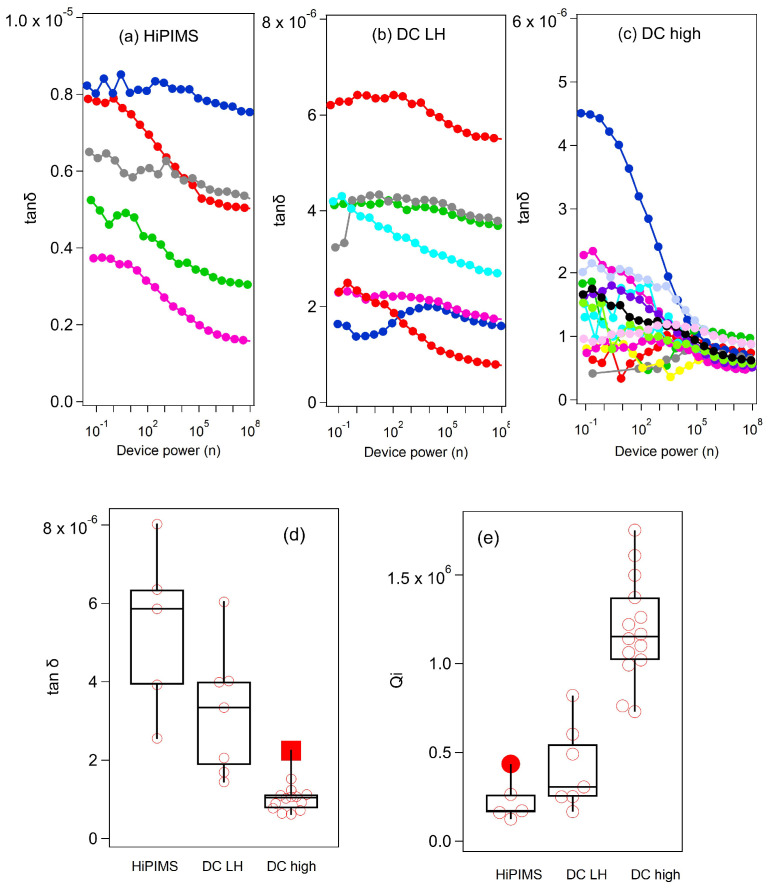
Power-dependent microwave characterization of Nb thin-film resonators. Loss tangent tan(δ) power spectra for three selected resonators made with (**a**) HiPIMS, (**b**) DC LH, (**c**) DC high samples. The device power (n) is converted to no. of photons operating at 5 GHz frequency. Different color traces are from different measurement scans. (**d**) Box plot of averaged loss tangent and (**e**) averaged internal $Q_{i}$ of Nb thin-film resonators. Each point in box plots is median value for one measurement, showing variation between different measurement sweeps. Filled points are outlier values far from median values.

Finally, the fs $\Delta R/R_{SC}$ dynamics under an applied magnetic field shown in Figure 3g reveals a clear correlation between non-equilibrium QPs and the quality factor of resonators fabricated by using different deposition methods. The thin-film Nb RRR = R(290 K)/R(10 K) and Nb resonator quality factor $Q_{i}$ are measured and presented in Figure 4. The Nb thin-film RRR values are 4.55 for HiPIMS, 6.66 for DC LH, and 6.53 for DC high. The low RRR value of HiPIMS is consistent with a smaller grain size compared to DC samples. Loss tangents tan(δ) are also obtained with $tan\left(\delta \right)=1/Q_{i}$ from measured $Q_{i}$. Figure 4 shows the loss tangent tan(δ) and $Q_{i}$ of RF resonators with different deposition methods. In Figure 4a–c, different colors represent different device power scans on the same samples. The power-dependent scans show high loss tangents at low power in general. The box plots in Figure 4d,e show median values for different scans. From the loss tangent box plot, HiPIMS has the highest loss, followed by DC LH and DC high. The median values of $Q_{i}$ are 0.1712 × $10^{6}$ for HiPIMS, 0.3064 × $10^{6}$ for DC LH, and 1.141 × $10^{6}$ for DC high. Because the $Q_{i}$ values were measured with the resonator only, the estimated upper limits $T_{1}$s for the qubit device are 13.86 μs for HiPIMS, 26.15 μs for DC LH, and 55.80 μs for DC high from $T_{1}=Q_{i}/\left(2\pi f\right)$ with an f = 5 GHz qubit operating frequency. These values align with the $\Delta R/R_{SC}$ results in Figure 3g, indicating that a smaller QP population and greater resistance to magnetic fields correlate with a higher $Q_{i}$ for Nb film samples. The Nb cavity cutout sample shows a significantly higher SRF cavity *Q* factor, consistent with this conclusion.

## 4. Conclusions

We measured femtosecond-resolved transient reflectivity in Nb thin-film resonators and SRF cavity cutout samples, examining non-equilibrium QP dynamics in the superconducting states across various temperatures and magnetic fields. The thermalized QP density and QP decay dynamics below the critical temperature display notably different behaviors based on the sample growth methods. Distinct differences in QP lifetime, density, and thermal diffusion among Nb samples are attributed to grain boundaries and defects.

The thermal diffusion contrasts significantly between thin films and bulk polycrystalline SRF cavities, with the SRF cavity exhibiting more efficient heat conduction. Magnetic-field-dependent measurements reveal clear behavioral distinctions between the two sample types, with the SRF sample showing stronger superconductivity against defects and faster QP relaxation characteristics that are highly favorable for applications requiring coherent quantum devices.

Within the thin-film samples, the DC high sample shows the lowest loss tangent in RF resonators, followed by DC LH, while HiPIMS has the highest loss tangent, which we attribute to grain boundary effects and grain size variations from different deposition methods. Magnetic-field-dependent thermal-equilibrium QP density measurements are consistent with this trend.

Our study further suggests that, in addition to a larger grain size and fewer defects to reduce TLS losses and QP pinning, Nb thin-film resonators fabricated on high-conductivity substrates such as sapphire could enhance transmon qubit performance. These findings emphasize the critical impact of fabrication techniques on the coherence and performance of Nb-based quantum devices, essential for applications in superconducting qubits and high-energy superconducting radio-frequency systems.

Finally, our results warrant a quantitative investigation of non-thermal pair breaking under a magnetic field, which could provide deeper insights into the quantum dynamics of superconducting vortex states.

## Figures and Tables

**Table 1:** Summary of Nb samples used in this paper. HiPIMS, DC high, and DC LH samples are sputter-coated on intrinsic Si wafers. Bulk SRF sample is cut out from SRF cavity.

Sample	Fabrication Method	Thickness	Average Grain Size	Substrate
HiPIMS	HiPIMS sputter	175 nm	44 nm	Si (001)
DC high	DC sputter (high power)	175 nm	69 nm	Si (001)
DC LH	DC sputter (low to high power)	175 nm (30 nm low, then 145 nm high)	65 nm	Si (001)
SRF	cavity cutout	2 mm	50 μm	bulk

## Data Availability

The original contributions presented in this study are included in the article. Further inquiries can be directed to the corresponding author.

## Abbreviations

The following abbreviations are used in this manuscript:

Al	aluminum
fs	femtosecond
HiPIMS	high-power impulse magnetron sputtering
HFP	high-frequency phonon
JJ	Josephson junction
Nb	niobium
QP	quasiparticle
R-T	Rothwarf and Taylor
SC	superconducting
SRF	superconducting radio frequency
TLS	two-level system

## Appendix A

### Appendix A.1

Skin depth δ=λ/(4πk) of Nb is plotted in Figure A1. Complex optical constants n+ik are from Palik’s handbook.

### Appendix A.2

The photoinduced QP density of the superconducting component, Q(T), of the Nb DC low–high sample is plotted in Figure A2 with a pump fluence of 3 μJ/cm^2^. Q(T) is the peak value obtained from ΔR/R in the superconducting state by subtracting from the average normal-state values measured at temperatures (T > $T_{c}$). Q(T) approaches 0 as the temperature gets closer to $T_{c}$. The overall shape of Q(T) indicates that the pump fluence is a weak perturbation region and the photoinduced QP density is smaller than the thermal-equilibrium QP density.

## References

[1] Siddiqi I. (2021). Engineering high-coherence superconducting qubits. Nat. Rev. Mater..

[2] Krantz P., Kjaergaard M., Yan F., Orlando T.P., Gustavsson S., Oliver W.D. (2019). A quantum engineer’s guide to superconducting qubits. Appl. Phys. Rev..

[3] Kjaergaard M., Schwartz M.E., Braumüller J., Krantz P., Wang J.I., Gustavsson S., Oliver W.D. (2020). Superconducting Qubits: Current State of Play. Annu. Rev. Condens. Matter. Phys..

[4] Premkumar A., Weil C., Hwang S., Jäck B., Place A.P., Waluyo I., Hunt A., Bisogni V., Pelliciari J., Barbour A. (2021). Microscopic relaxation channels in materials for superconducting qubits. Commun. Mater..

[5] Blais A., Huang R.S., Wallraff A., Girvin S.M., Schoelkopf R.J. (2004). Cavity quantum electrodynamics for superconducting electrical circuits: An architecture for quantum computation. Phys. Rev. A.

[6] Oliver W.D., Welander P.B. (2013). Materials in superconducting quantum bits. MRS Bull..

[7] Blais A., Grimsmo A.L., Girvin S.M., Wallraff A. (2021). Circuit quantum electrodynamics. Rev. Mod. Phys..

[8] Wallraff A., Schuster D.I., Blais A., Frunzio L., Huang R.S., Majer J., Kumar S., Girvin S.M., Schoelkopf R.J. (2004). Strong coupling of a single photon to a superconducting qubit using circuit quantum electrodynamics. Nature.

[9] Harrelson T.F., Sheridan E., Kennedy E., Vinson J., N’Diaye A.T., Altoé M.V.P., Schwartzberg A., Siddiqi I., Ogletree D.F., Scott M.C. (2021). Elucidating the local atomic and electronic structure of amorphous oxidized superconducting niobium films. Appl. Phys. Lett..

[10] Carroll M., Rosenblatt S., Jurcevic P., Lauer I., Kandala A. (2022). Dynamics of superconducting qubit relaxation times. npj Quantum Inf..

[11] Zhang E.J., Srinivasan S., Sundaresan N., Bogorin D.F., Martin Y., Hertzberg J.B., Timmerwilke J., Pritchett E.J., Yau J.B., Wang C. (2022). High-performance superconducting quantum processors via laser annealing of transmon qubit. Sci. Adv..

[12] Bravyi S., Dial O., Gambetta J.M., Gil D., Nazario Z. (2022). The future of quantum computing with superconducting qubits. J. Appl. Phys..

[13] Smirnov N.S., Krivko E.A., Solovyova A.A., Ivanov A.I., Rodionov I.A. (2024). Wiring surface loss of a superconducting transmon qubit. Sci. Rep..

[14] Verjauw J., Acharya R., Van Damme J., Ivanov T., Lozano D.P., Mohiyaddin F.A., Wan D., Jussot J., Vadiraj A.M., Mongillo M. (2022). Path toward manufacturable superconducting qubits with relaxation times exceeding 0.1 ms. npj Quantum Inf..

[15] De Graaf S.E., Faoro L., Ioffe L.B., Mahashabde S., Burnett J.J., Lindström T., Kubatkin S.E., Danilov A.V., Tzalenchuk A.Y. (2020). Two-level systems in superconducting quantum devices due to trapped quasiparticles. Sci. Adv..

[16] Müller C., Cole J.H., Lisenfeld J. (2019). Towards understanding two-level-systems in amorphous solids: Insights from quantum circuits. Rep. Prog. Phys..

[17] Ristè D., Bultink C.C., Tiggelman M.J., Schouten R.N., Lehnert K.W., DiCarlo L. (2013). Millisecond charge-parity fluctuations and induced decoherence in a superconducting transmon qubit. Nat. Commun..

[18] Vepsäläinen A.P., Karamlou A.H., Orrell J.L., Dogra A.S., Loer B., Vasconcelos F., Kim D.K., Melville A.J., Niedzielski B.M., Yoder J.L. (2020). Impact of ionizing radiation on superconducting qubit coherence. Nature.

[19] Rothwarf A., Taylor B.N. (1967). Measurement of Recombination Lifetimes in Superconductors. Phys. Rev. Lett..

[20] Kabanov V.V., Demsar J., Mihailovic D. (2005). Kinetics of a Superconductor Excited with a Femtosecond Optical Pulse. Phys. Rev. Lett..

[21] Stojchevska L., Kusar P., Mertelj T., Kabanov V.V., Toda Y., Yao X., Mihailovic D. (2011). Mechanisms of nonthermal destruction of the superconducting state and melting of the charge-density-wave state by femtosecond laser pulses. Phys. Rev. B.

[22] Federici J.F., Greene B.I., Saeta P.N., Dykaar D.R., Sharifi F., Dynes R.C. (1992). Direct picosecond measurement of photoinduced Cooper-pair breaking in lead. Phys. Rev. B.

[23] Yang X., Zhao X., Vaswani C., Sundahl C., Song B., Yao Y., Cheng D., Liu Z., Orth P.P., Mootz M. (2019). Ultrafast nonthermal terahertz electrodynamics and possible quantum energy transfer in the Nb3Sn superconductor. Phys. Rev. B.

[24] Cheng B., Cheng D., Lee K., Luo L., Chen Z., Lee Y., Wang B.Y., Mootz M., Perakis I.E., Shen Z.X. (2024). Evidence for d–wave superconductivity of infinite-layer nickelates from low-energy electrodynamics. Nat. Mater..

[25] Rothwarf A., Sai-Halasz G.A., Langenberg D.N. (1974). Quasiparticle Lifetimes and Microwave Response in Nonequilibrium Superconductors. Phys. Rev. Lett..

[26] Chang J.-J., Scalapino D.J. (1977). Kinetic-equation approach to nonequilibrium superconductivity. Phys. Rev. B.

[27] Gedik N., Blake P., Spitzer R.C., Orenstein J., Liang R., Bonn D.A., Hardy W.N. (2004). Single-quasiparticle stability and quasiparticle-pair decay in YBa_2_Cu_3_O_6.5_. Phys. Rev. B.

[28] Hinton J.P., Thewalt E., Alpichshev Z., Mahmood F., Koralek J.D., Chan M.K., Veit M.J., Dorow C.J., Barišić N., Kemper A.F. (2016). The rate of quasiparticle recombination probes the onset of coherence in cuprate superconductors. Sci. Rep..

[29] Chia E.E.M., Talbayev D., Zhu J., Yuan H.Q., Park T., Thompson J.D., Panagopoulos C., Chen G.F., Luo J.L., Wang N.L. (2010). Ultrafast Pump-Probe Study of Phase Separation and Competing Orders in the Underdoped (Ba,K)Fe_2_As_2_ Superconductor. Phys. Rev. Lett..

[30] Lin K.H., Wang K.J., Chang C.C., Wen Y.C., Lv B., Chu C.W., Wu M.K. (2016). Ultrafast dynamics of quasiparticles and coherent acoustic phonons in slightly underdoped (BaK)Fe_2_As_2_. Sci. Rep..

[31] Yang X., Luo L., Mootz M., Patz A., Bud’ko S.L., Canfield P.C., Perakis I.E., Wang J. (2018). Nonequilibrium Pair Breaking in Ba(Fe_1−x_Cox)_2_As_2_ Superconductors: Evidence for Formation of a Excitonic State. Phys. Rev. Lett..

[32] Oh J.S., Kopas C.J., Marshall J., Fang X., Joshi K.R., Datta A., Ghimire S., Park J.M., Kim R., Setiawan D. (2024). Correlating Deposition Method, Microstructure, and Performance of Nb/Si-based Superconducting Coplanar Waveguide Resonators. Acta Mater..

[33] Checchin M., Martinello M., Grassellino A., Aderhold S., Chandrasekaran S.K., Melnychuk O.S., Posen S., Romanenko A., Sergatskov D.A. (2018). Frequency dependence of trapped flux sensitivity in SRF cavities. Appl. Phys. Lett..

[34] Cheng D., Song B., Kang J.-H., Sundahl C., Edgeton A.L., Luo L., Park J.-M., Collantes Y.G., Hellstrom E.E., Mootz M. (2023). Study of Elastic and Structural Properties of BaFe_2_As_2_ Ultrathin Film Using Picosecond Ultrasonics. Materials.

[35] Palik E.D. (1991). Handbook of Optical Constants of Solids.

